# Site-Specific Incorporation of Functional Components into RNA by an Unnatural Base Pair Transcription System

**DOI:** 10.3390/molecules17032855

**Published:** 2012-03-07

**Authors:** Nobuyuki Morohashi, Michiko Kimoto, Akira Sato, Rie Kawai, Ichiro Hirao

**Affiliations:** 1 TagCyx Biotechnologies, 1-6-126 Suehiro-cho, Tsurumi-ku, Yokohama, Kanagawa 230-0045, Japan; E-Mails: morohash@chem.meisei-u.ac.jp (N.M.); michikoh@riken.jp (M.K.); rkawai@he.netyou.jp (R.K.); 2 RIKEN Systems and Structural Biology Center (SSBC), 1-7-22 Suehiro-cho, Tsurumi-ku, Yokohama, Kanagawa 230-0045, Japan; E-Mail: asato@riken.jp

**Keywords:** unnatural base pair, transcription, T7 RNA polymerase

## Abstract

Toward the expansion of the genetic alphabet, an unnatural base pair between 7-(2-thienyl)imidazo[4,5-*b*]pyridine (**Ds**) and pyrrole-2-carbaldehyde (**Pa**) functions as a third base pair in replication and transcription, and provides a useful tool for the site-specific, enzymatic incorporation of functional components into nucleic acids. We have synthesized several modified-**Pa** substrates, such as alkylamino-, biotin-, TAMRA-, FAM-, and digoxigenin-linked **Pa**TPs, and examined their transcription by T7 RNA polymerase using **Ds**-containing DNA templates with various sequences. The **Pa** substrates modified with relatively small functional groups, such as alkylamino and biotin, were efficiently incorporated into RNA transcripts at the internal positions, except for those less than 10 bases from the 3′-terminus. We found that the efficient incorporation into a position close to the 3′-terminus of a transcript depended on the natural base contexts neighboring the unnatural base, and that pyrimidine-**Ds**-pyrimidine sequences in templates were generally favorable, relative to purine-**Ds**-purine sequences. The unnatural base pair transcription system provides a method for the site-specific functionalization of large RNA molecules.

## 1. Introduction

Expansion of the genetic alphabet of DNA by an unnatural base pair system could be a powerful tool for the site-specific incorporation of extra functional components into nucleic acids and proteins. The realization of this genetic expansion system requires the development of an unnatural base pair that functions in biological systems, such as replication, transcription, and translation, with highly exclusive selectivity as a third base pair, along with the natural A–T and G–C pairs. Researchers are attempting to create expanded systems, and many unnatural base pairs have been designed and tested in *in vitro* biological systems [1,2,3,4,5]. Among them, some unnatural base pairs have exhibited high selectivity as a third base pair in PCR amplification and/or transcription [6,7,8,9,10,11,12,13,14,15,16,17,18,19].

In the course of our research, we developed several unnatural base pairs, such as those between 2-amino-6-thienylpurine (**s**) and 2-oxopyridine (**y**) [13,20,21,22], 7-(2-thienyl)-imidazo[4,5-*b*]pyridine (**Ds**) and pyrrole-2-carbaldehyde (**Pa**) [6], **Ds** and 2-nitropyrrole (**Pn**) [23], and **Ds** and 2-nitro-4-propynylpyrrole (**Px**) [7,8], toward practical use in DNA/RNA-based biotechnologies. The **Ds**–**Px** pair exhibits the highest selectivity and efficiency in PCR amplification, and the **Ds**–**Pa** pair is suitable for the site-specific incorporation of **Pa** and its modified bases, as well as **Ds**, into RNA by transcription. The substrates of modified-**Pa** bases, in which several functional groups are attached to position 4 of **Pa** via a propynyl linker, can be site-specifically incorporated into RNA, opposite **Ds** in templates, by T7 RNA polymerase. For transcription involving the **Ds**–**Pa** pair, DNA templates containing **Ds** are prepared by PCR amplification using the **Ds**–**Px** pair. Thus, the combination of the **Ds**–**Px** and **Ds**–**Pa** pairs would be useful for the direct site-specific modification of long RNA molecules by T7 transcription.

To provide a highly versatile method for generating functional RNA molecules with an increased number of components by the **Ds**–**Pa** pair system, we here report the site-specific incorporation of several modified-**Pa** substrates, linked with alkylamino, biotin, or fluorescent groups, into RNA transcripts (from 17-mer to 48-mer) using DNA templates containing **Ds** with different sequence contexts and lengths. We previously reported that the PCR amplification efficiency involving the **Ds**–**Px** pair depends on the natural base contexts around the unnatural base. For example, we found that the efficiency of pyrimidine-**Ds**-pyrimidine sequences in both the template and complementary growing strands is higher than that of purine-**Ds**-purine sequences [8]. In this report, we examined whether the sequence dependency is also observed in transcription involving the **Ds**–**Pa** pair, as in the case of PCR amplification involving the **Ds**–**Px** pair. We found that **Pa** substrates modified with relatively small functional groups, such as alkylamino and biotin, were efficiently and selectively incorporated into RNA transcripts at the internal positions, except for those less than 10 bases from the 3′-terminus, without any sequence dependency. Although the incorporation efficiency and selectivity were reduced for **Pa** substrates modified with relatively large functional groups, such as TAMRA, FAM, and digoxigenin, their incorporation can be practically used for the site-specific labeling of RNA transcripts. In addition, the efficiency of the site-specific incorporation of all modified-**Pa** substrates into a position close to the 3′-terminus of a transcript significantly depends on the natural base sequence contexts around the unnatural base in templates. In this case, the sequence dependency is similar to that in replication involving the **Ds**–**Px** pair. Overall, the specific transcription using the **Ds**–**Pa** pair could be a useful tool for the site-specific functionalization of long RNA molecules, based on a fundamental understanding of the characteristics of the **Ds**–**Pa** pair in transcription.

## 2. Results and Discussion

### 2.1. Chemical Syntheses of Modified-**Pa** Triphosphates (Modified-**Pa**TPs) and **Ds**-Containing DNA Templates

**Figure 1:**
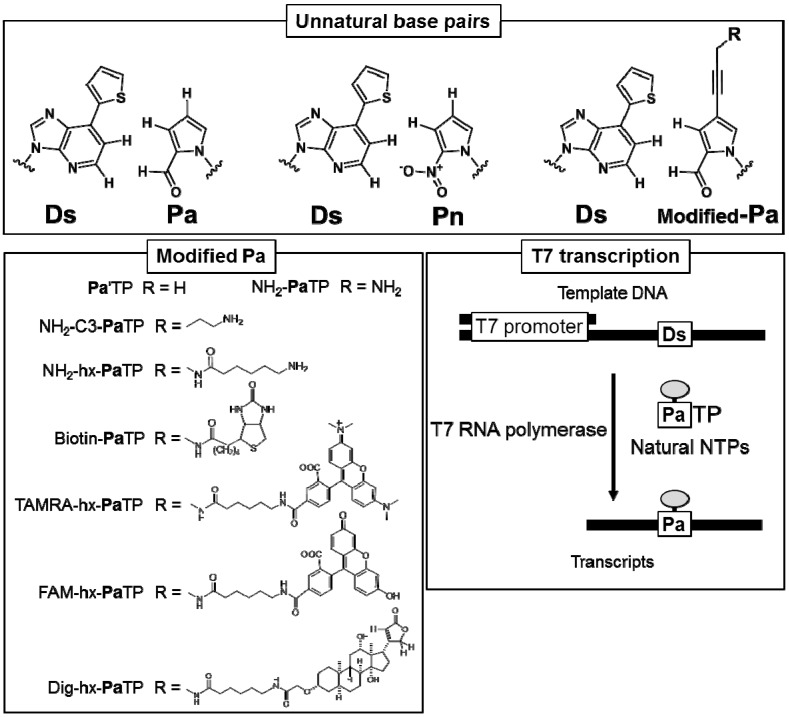
Chemical structures of unnatural base pairs used for T7 transcription in this study. A variety of substituent groups can be attached to the **Pa** base moiety. The scheme of T7 transcription mediated by the unnatural **Ds**–**Pa** pair is shown (lower right).

To examine the site-specific incorporation efficiency of a variety of modified-**Pa** triphosphate substrates (modified-**Pa**TPs) into RNA transcripts by the **Ds**–**Pa** pair system, we chemically synthesized several modified-**Pa**TPs with a variety of functional groups, such as alkylamino groups, biotin, digoxigenin (Dig), and fluorescent dyes (FAM and TAMRA), which are attached to position 4 of the **Pa** base via a propynyl linker (Figure 1).

**Scheme 1:**
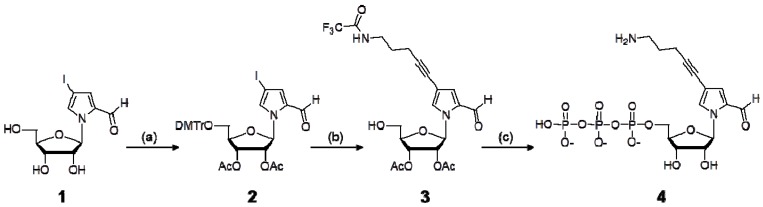
Synthesis of the nucleoside 5′-triphosphate of NH_2_-C3-**Pa**.

**Scheme 2:**
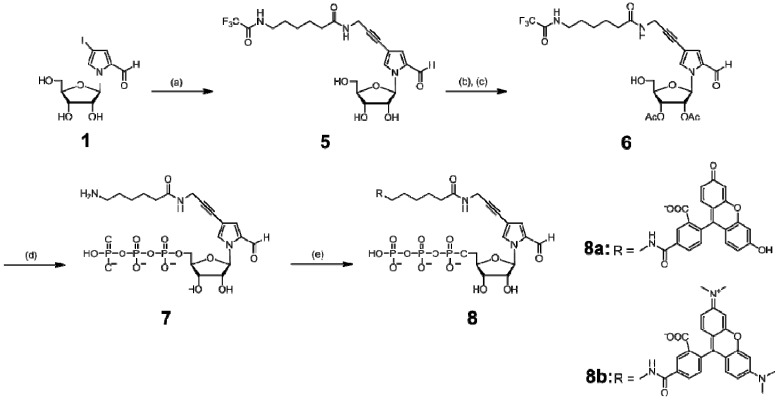
Synthesis of the nucleoside 5′-triphosphates of TAMRA- and FAM-hx-**Pa**.

The modified-**Pa** substrates of **Pa′**TP [6], NH_2_-**Pa**TP [6], NH_2_-C3-**Pa**TP, and NH_2_-hx-**Pa**TP were prepared by palladium-mediated cross-coupling of a 4-iodopyrrole-2-carbaldehyde nucleoside with the functional groups of 1-propyne, 3-(dichloroacetamido)-1-propyne, 5-trifluoroacetamide-1-pentyne, and *N*-(2-propynyl)-6-trifluoroacetamidohexanamide, respectively (Scheme 1 and Scheme 2).

**Scheme 3:**
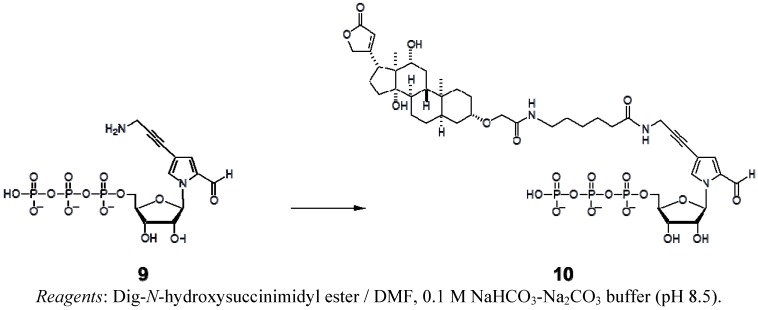
Synthesis of the nucleoside 5′-triphosphate of Dig-hx-**Pa**.

These nucleoside derivatives were then converted to the triphosphate substrates (bearing free amino groups for alkylamino-**Pa**TPs) by Eckstein’s method. Biotin-**Pa**TP [6] and Dig-hx-**Pa**TP were prepared by the treatment of NH_2_-**Pa**TP with biotin-*N*-hydroxysuccinimide and digoxigenin-3-*O*-methylcarbonyl-ε-aminocaproic acid-*N*-hydroxysuccinimide ester, respectively (Scheme 3). TAMRA-hx-**Pa**TP and FAM-hx-**Pa**TP were prepared by the treatment of NH_2_-hx-**Pa**TP with 5-carboxytetramethylrhodamine *N*-hydroxysuccinimidyl ester and 5-carboxyfluorescein *N*-hydroxy-succinimidyl ester, respectively (Scheme 2). The relatively large groups, such as TAMRA, FAM, and digoxigenin, were attached to the 4-aminopropynyl **Pa** base via an aminohexyl linker, to reduce the steric hindrance in the nucleic acid and polymerase complexes during transcription. All modified-**Pa** triphosphates were purified by DEAE Sephadex column chromatography and C18 HPLC. Their structures were identified by ^1^H- and ^31^P-NMR and mass spectrometry.

DNA templates containing **Ds** were prepared by standard phosphoramidite chemistry, using a DNA synthesizer [6]. The synthesized DNA fragments were purified by gel electrophoresis. Full-length template strands (from 35-mer to 65-mer) containing one **Ds** base were hybridized with a 21-mer non-template strand including the T7 promoter region for transcription (Figure 1).

### 2.2. T7 Transcription for 48-Mer Transcripts Containing Modified-**Pa** at Internal Positions

**Figure 2:**
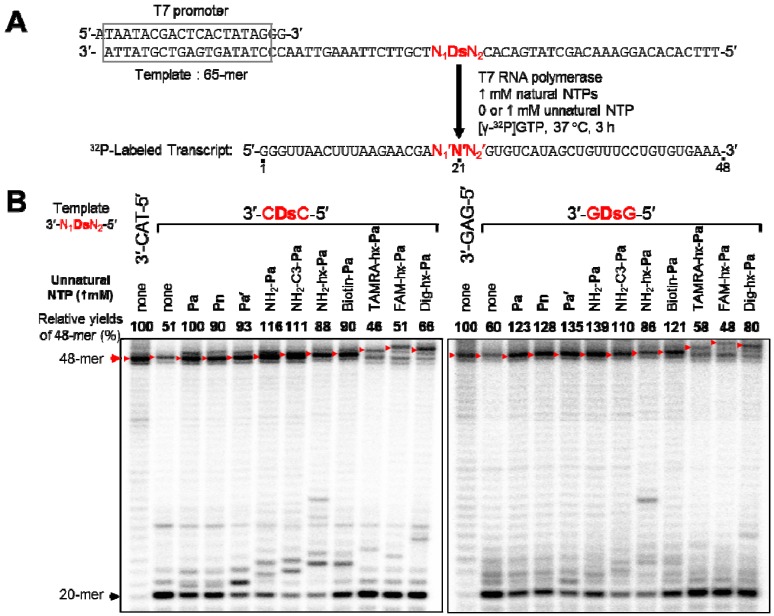
T7 transcription mediated by the **Ds**–**Pa** pair for 48-mer transcripts. (**a**) Scheme of the experiments. (**b**) Gel electrophoresis of transcripts generated from a template containing a 3′-C**Ds**C-5′ or 3′-G**D**sG-5′ sequence with natural NTPs (1 mM) and each modified-**Pa**TP or **Pn**TP (1 mM). Transcripts were labeled at their 5′-termini with [γ-^32^P]GTP. The positions of the full-length 48-mer products are indicated by red arrows. Due to the differences in the substituent groups attached to the **Pa** base moiety, the mobilities of the transcripts containing modified-**Pa** differed. The relative yield of each full-length transcript was determined by comparison to the yield of the native transcript from the template consisting of the natural bases only (3′-CAT-5′). The black arrow indicates the position of 20-mer transcripts, which paused at the previous position of **Ds** in the template. The experiments were independently repeated three times, and representative data are shown.

**Figure 3:**
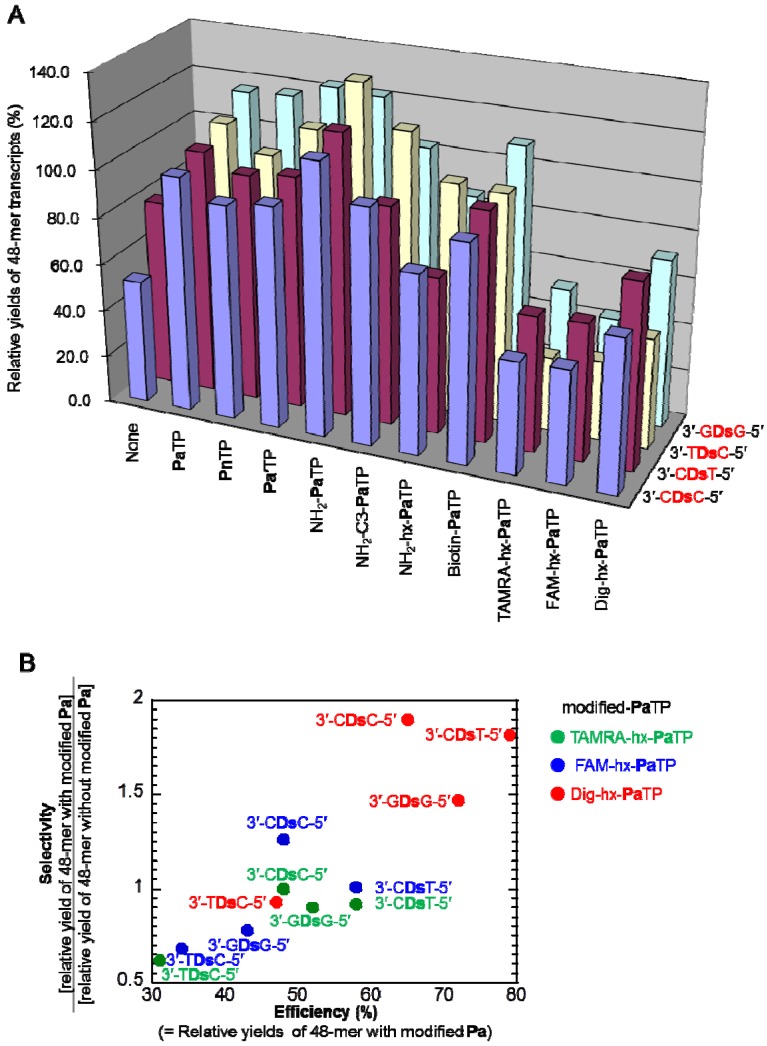
Efficiency and selectivity analysis of the 48-mer transcripts. **(a)** Plot of relative yields of the 48-mer transcripts from templates containing 3′-C**Ds**C-5′, 3′-C**Ds**T-5′, 3′-T**Ds**C-5′, or 3′-G**Ds**G-5′, in the presence of each modified-**Pa**TP or **Pn**TP. The relative yield of each full-length transcript was determined by comparison to the yield of the native transcript from the template consisting of the natural bases only (3′-N_1_**Ds**N_2_-5′ = 3′-CAT-5′ for 3′-C**Ds**C-5′, 3′-C**Ds**T-5′, 3′-T**Ds**C-5′; 3′-N_1_**Ds**N_2_-5′ = 3′-GAG-5′ for 3′-G**Ds**G-5′). The values were averaged from three data sets. **(b)** Correlation between the incorporation efficiency and selectivity of **Pa**TPs modified with large molecules in T7 transcription. The selectivity was determined as the ratio of the relative yield of the 48-mer transcripts with and without modified-**Pa** bases (TAMRA-hx-**Pa**, FAM-hx-**Pa**, and Dig-hx-**Pa**) from the gel analysis of transcripts.

We first examined the incorporation efficiency of each modified-**Pa**TP, as well as **Pn**TP, in transcription by T7 RNA polymerase, using 65-mer DNA templates containing one **Ds** base, in which **Ds** was located at the complementary site corresponding to position 21 in the 48-mer transcripts (Figure 2A). To investigate the natural base sequence dependency around the **Ds** base in templates, we used four templates containing **Ds** in different sequence contexts, 3′-C**Ds**C-5′, 3′-C**Ds**T-5′, 3′-T**Ds**C-5′, and 3′-G**Ds**G-5′, as well as control templates, 3′-CAT-5′ and 3′-GAG-5′, comprising only the natural bases. Transcription was performed in the presence of 1 mM natural base NTPs, 0 or 1 mM modified-**Pa**TP or **Pn**TP, [γ-^32^P] GTP, DNA templates, and T7 RNA polymerase, at 37 °C for 3 h. After transcription, the 5′-labeled transcripts were analyzed by gel electrophoresis (Figure 2B), and the relative yields of the full-length transcripts (48-mer) were determined by comparison to the yield of transcription using the control template (Figure 2B and Figure 3A). On the gel, the mobilities of the full-length transcripts differed, depending on the modifications of the **Pa** base. The incorporation of **Pa**TPs modified with large molecules (TAMRA-hx-**Pa**TP, FAM-hx-**Pa**TP, and Dig-hx-**Pa**TP) reduced the mobility of the transcripts on the gel, and the incorporation of TAMRA-hx-**Pa** or FAM-hx-**Pa** into the full-length transcripts was confirmed by monitoring their fluorescence with a bio-imager, FLA-7000 (data not shown). Truncated products (20-mer), which resulted from pausing before the unnatural base position, were also detected. All of the unnatural base substrates, **Pa**TP, **Pn**TP, and modified-**Pa**TPs, except for the **Pa**TPs modified with the large molecules, were efficiently incorporated into RNA by T7 transcription, and the transcription efficiency (86−139%) was independent of both the sequence contexts and the amounts of the truncated products (20-mer). Furthermore, we found that **Pn**TP, with a 2-nitro group instead of the 2-aldehyde group of **Pa**, is also a favorable substrate as a pairing partner of **Ds** in transcription, as shown in Figure 2 and Figure 3A.

The **Pa**TPs modified with the large molecules were less effective, and the transcription yields were reduced by nearly half. In addition, the **Pa**TPs modified with the large molecules exhibited reduced incorporation selectivity opposite **Ds** in templates, and the full-length transcripts without modified-**Pa** also appeared on the gel. We determined the incorporation selectivities of TAMRA-hx-**Pa**TP, FAM-hx-**Pa**TP, and Dig-hx-**Pa**TP, from the ratios of the amounts of the full-length transcripts with modified-**Pa** to those without modified-**Pa** (Figure 3B). 

Since the bands corresponding to the full-length transcripts containing these modified-**Pa** were shifted on the gel, the incorporation selectivity of each modified-**Pa** could be determined by comparing the band densities between the full-length transcripts with and without the modified-**Pa** bases. Although the incorporation selectivities of the **Pa**TPs modified with the large molecules were relatively low, the selectivity of each modified-**Pa** correlated well with its incorporation efficiency into RNA. Depending on the sequence context of the templates, more efficient incorporation tended to lead to more selective incorporations. Next, we determined the incorporation position of Biotin-**Pa** in the full-length transcripts. We previously reported the high selectivity of the **Pa**TP and Biotin-**Pa**TP incorporations opposite **Ds** by transcription, using DNA templates containing 3′-C**Ds**T-5′ and 3′-A**Ds**T-5′ sequences, respectively [6]. 

**Figure 4:**
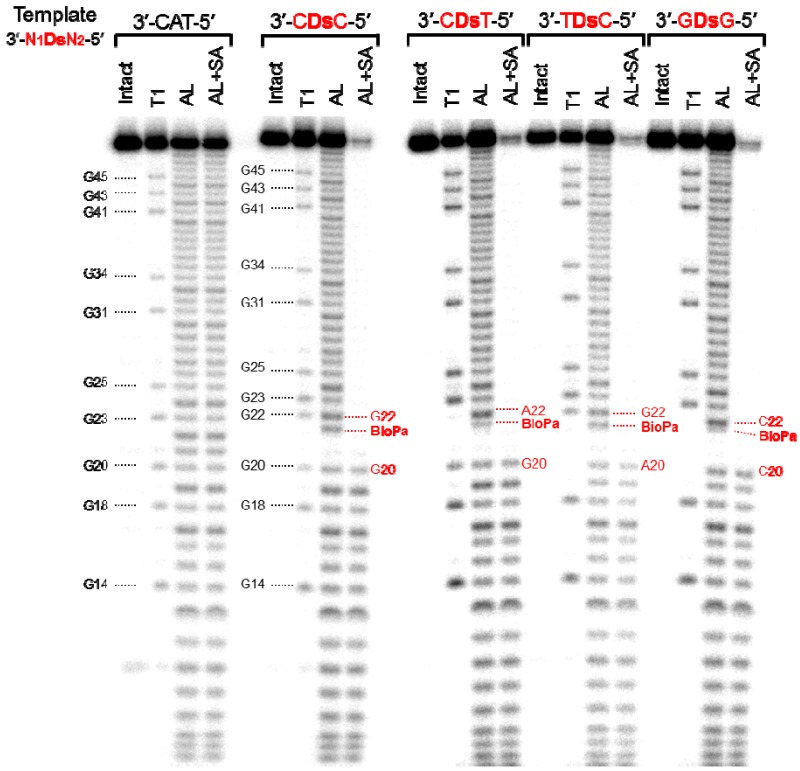
Sequence analysis of the 48-mer transcripts containing Biotin-**Pa** or U at position 21. The 5′ ^3^^2^P-labeled transcripts were partially digested with either RNase T1 (T1) or with alkali (AL). A portion of the partially alkali-digested transcripts was treated with streptavidin magnetic beads, to capture the RNA fragments containing Biotin-**Pa** (AL+SA). Each digested fragment was analyzed on a 10% polyacrylamide gel containing 7 M urea.

Therefore, we confirmed the incorporation positions of Biotin-**Pa** in the full-length transcripts, obtained by transcription using DNA templates with four different sequence contexts, 3′-C**Ds**C-5′, 3′-C**Ds**T-5′, 3′-T**Ds**C-5′, and 3′-G**Ds**G-5′. The ^32^P-labeled full-length transcripts were partially digested by either alkali or RNase T1, and the digested products were incubated with streptavidin magnetic beads. This treatment separated the Biotin-**Pa**-containing fragments from the digested products, and the remaining fragments without Biotin-**Pa** were analyzed by gel electrophoresis (Figure 4). By comparing the sequencing ladder patterns obtained by the alkali and RNase T1 treatments, the position of Biotin-**Pa** incorporation was determined from the initial disappearance position of the streptavidin-treated ladders.

On the patterns obtained by the streptavidin treatment of all four transcripts with different sequences, the ladders corresponding to the fragments larger than 21-mer were almost undetectable. Based on the disappearance ratio of the ladders, more than 90% of Biotin-**Pa** was incorporated at the desired position in the transcripts. In the alkali-digested products without the streptavidin treatment, the ladders corresponding to the fragments larger than 21-mer were largely shifted on the gel. From these results, we concluded that Biotin-**Pa**TP was site-specifically incorporated into the transcripts at position 21, opposite **Ds** in the templates. The incorporation selectivity of Biotin-**Pa**TP opposite **Ds** was more than 90%, and was independent of the sequence context.

### 2.3. T7 Transcription for Transcripts Containing **Pa** at Different Positions from the 3′-Terminus

We next examined how the position of **Pa** incorporation, especially close to the 3′-terminus, in transcripts affects the incorporation efficiency. We previously reported that transcription efficiency involving the **Ds**–**Pa** pairing decreased when using short DNA templates (such as 35-mer) [6].

**Figure 5:**
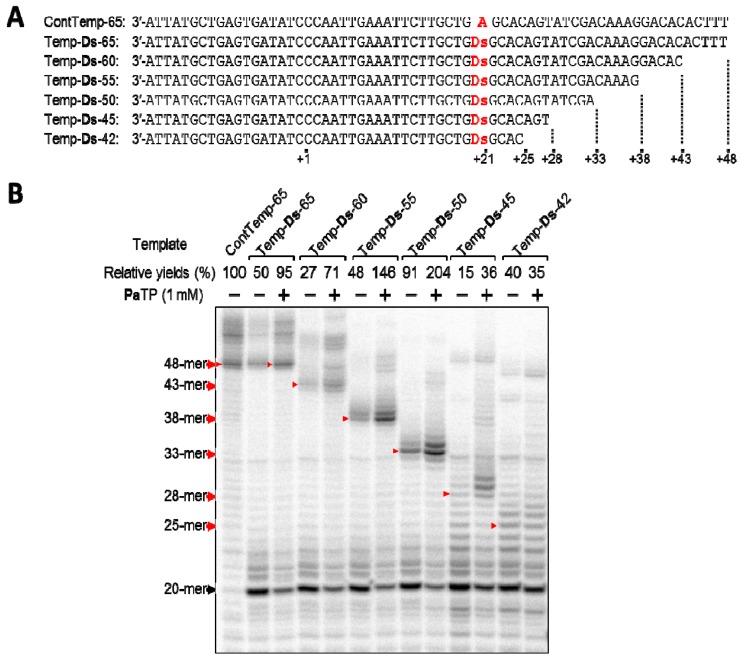
T7 transcription mediated by the **Ds**–**Pa** pair from DNA templates containing **Ds**, with different lengths after the unnatural base position. (**a**) Sequences of templates used in this study. (b) Gel electrophoresis of transcripts from different length templates with natural NTPs (1 mM) and in the presence or absence of **Pa**TP (1 mM). Transcripts were labeled at their 5′-termini with [γ-^32^P]GTP. The positions of the full-length products are indicated by red arrows. The relative yield of each full-length transcript was determined by comparison to the yield of the native transcript from the template consisting of the natural bases only. The experiments were independently repeated three times, and representative data are shown.

Thus, we prepared a series of DNA templates (42**–**65-mer), which were shortened stepwise from the terminus, and examined T7 transcription using the templates (Figure 5). For the experiments, we chose the DNA template containing the 3′-G**Ds**G-5′ sequence, which was expected to be an inferior template based on our replication experiments involving related unnatural base pairs [7,8]. Using the DNA templates and **Pa**TP, we compared the yields of transcripts (25-, 28-, 33-, 38-, 43-, and 48-mer). 

The full-length transcripts (48-, 43-, 38-, and 33-mer) obtained from 65-, 60-, 55-, and 50-mer templates were observed as fairly clear bands on the gel, and the relative yields of these transcripts were more than 71%, as compared to that using the control template (ContTemp-65) with natural NTPs. In contrast, the full-length transcripts (28-mer and 25-mer) from the 45- and 42-mer templates were difficult to discern as a single main band on the gel, and the transcription yields were also reduced, to 35–36%. When using the 45-mer and 42-mer templates, several ladders, which were larger than the full-length transcripts, appeared on the gel. Although we still do not know how the ladders were generated, this might be an intrinsic problem caused by transcript-transcript or transcript-template interactions, depending on the natural base sequence contexts [24] or the instability of the complex between the template, resulting transcript, substrate, and T7 RNA polymerase. Even allowing for this problem, the incorporation of **Pa** at a position less than 12-bases from the 3′-terminus of the transcripts significantly reduces the transcription efficiency involving the **Ds**–**Pa** pairing, when using templates with the inferior 3′-G**Ds**G-5′ sequence. These results compelled us to examine how the sequence contexts in templates affect the transcription efficiency, when the **Pa** incorporation site is close to the 3′-terminus in the transcripts.

### 2.4. **Pa** Incorporation into the 3′-Region of Transcripts with Different Sequence Contexts around the Unnatural Base

**Figure 6:**
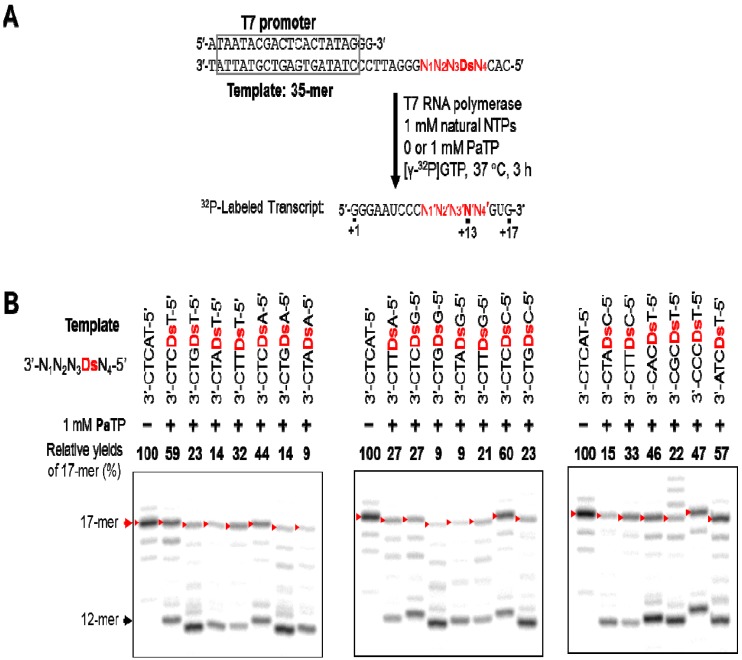
T7 transcription mediated by the **Ds**–**Pa** pair for 17-mer transcripts. (**a**) Scheme of the experiments. (**b**) Gel electrophoresis of transcripts generated from templates with different sequences around **Ds**, with natural NTPs (1 mM), in the presence or absence of **Pa**TP (1 mM). Transcripts were labeled at their 5′-termini with [γ-^32^P]GTP. The positions of the full-length 17-mer products are indicated by red arrows. Due to the different sequence contexts, the mobilities of the transcripts differed. The relative yield of each full-length transcript was determined by comparison to the yield of the native transcript from the template consisting of the natural bases only. The black arrow indicates the approximate position of the 12-mer transcripts, which paused at the previous position of **Ds** in the template. The experiments were independently repeated three times, and representative data are shown.

**Figure 7:**
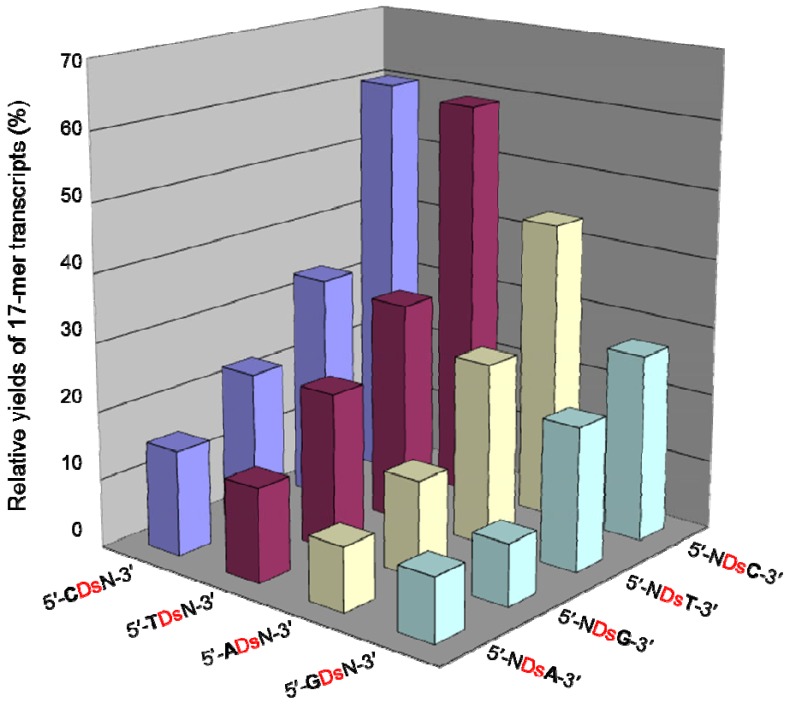
Plot of the relative yields of the 17-mer transcripts from the template containing a 3′-N_1_**Ds**N_2_-5′ sequence (N_1_, N_2_ = A, G, C, or T), in the presence of **Pa**TP. The relative yield of each full-length transcript was determined by comparison to the yield of the native transcript from the template consisting of the natural bases only. The values were averaged from three data sets.

We then examined the transcription efficiency using short DNA templates (35-mer) containing 20 sequence combinations, including all 3′-CTN**Ds**N-5′ sequences, where N = A, G, C or T, as well as 3′-CAC**Ds**T-5′, 3′-CGC**Ds**T-5′, 3′-CCC**Ds**T-5′, and 3′-ATC**Ds**T-5′. RNA fragments (17-mer) containing **Pa** at position 13, 5-bases from the 3′-terminus, were transcribed as full-length products, using the same method described above. The representative gel data are displayed in Figure 6, and the transcription efficiencies for all 16 templates are summarized in Figure 7.

**Figure 8:**
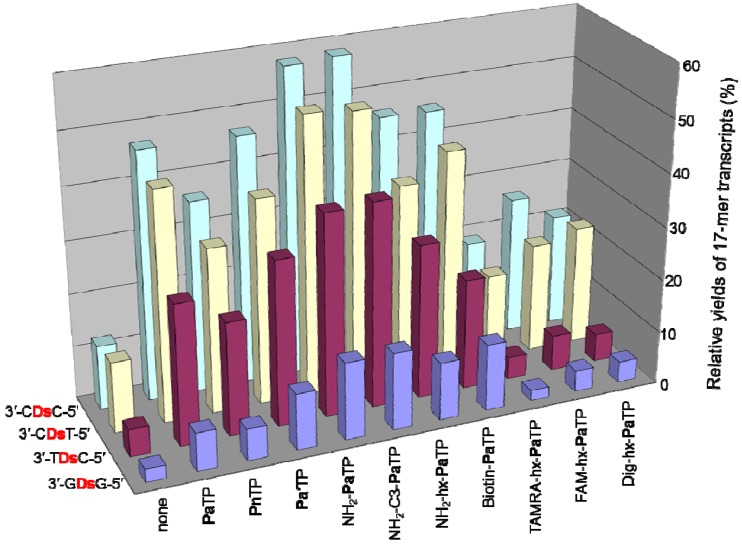
Plot of the relative yields of the 17-mer transcripts from templates with different sequences around **Ds**, with natural NTPs (1 mM), in the presence of each modified-**Pa**TP or **Pn**TP (1 mM). The relative yield of each full-length transcript was determined by comparison to the yield of the native transcript from the template consisting of the natural bases only (3′-N_1_**Ds**N_2_-5′ = 3′-CAT-5′ for ′-C**Ds**C-5′, 3′-C**Ds**T-5′, 3′-T**Ds**C-5′; 3′-N_1_**Ds**N_2_-5′ = 3′-GAG-5′ for ′-G**Ds**G-5′). The values were averaged from three to four data sets.

We found that the adjacent natural base contexts around the unnatural base significantly affected the transcription efficiency involving the **Ds**–**Pa** pairing, when the **Pa** incorporation site is close to the 3′-terminus of the transcript. The order of the effective sequences was 3′-C**Ds**C-5′ ≈ 3′-C**Ds**T-5′ > 3′-C**Ds**A-5′ > 3′-T**Ds**C-5′ ≈ 3′-T**Ds**T-5′ > 3′-T**Ds**A-5′ ≈ 3′-C**Ds**G-5′ ≈ 3′-G**Ds**C-5′ ≈ 3′-G**Ds**T-5′ > 3′-T**Ds**G-5′ > 3′-A**Ds**C-5′ ≈ 3′-A**Ds**T-5′ ≈ 3′-G**Ds**A-5′ > 3′-A**Ds**A-5′ ≈ 3′-A**Ds**G-5′ ≈ 3′-G**Ds**G-5′ (Figure 7). Thus, 3′-pyrimidine-**Ds**-pyrimidine-5′ is generally better than 3′-purine-**Ds**-purine-5′ for efficient transcription. Even using the short templates, the 3′-C**Ds**C-5′ and 3′-C**Ds**T-5′ sequence contexts generated relatively high yields (60%) of the 17-mer transcripts. As shown in Figure 6, templates containing 3′-CTC**Ds**T-5′, 3′-CAC**Ds**T-5′, 3′-CCC**Ds**T-5′, and 3′-ATC**Ds**T-5′ sequences provided similar transcription efficiency (relative yields of the 17-mer transcripts = 46–59%). These templates have the same 3′-C**Ds**T-5′ sequence, and thus, the adjacent natural bases around the unnatural base significantly affect the transcription efficiency. The template containing a 3′-CGC**Ds**T-5′ sequence was the only exception, and the relative yield of the 17-mer transcript was 22%. However, in this case, some transcripts (longer than 17-mer) appeared on the gel. These products might be obtained by unusual interactions between the transcripts and templates [24], and thus the low yield of the 17-mer transcript is due to the by-products and is not related to the **Ds**–**Pa** pairing in transcription. We also determined the incorporation efficiencies of **Pa**TP, **Pn**TP, and eight modified-**Pa**TPs using short DNA templates (35-mer) containing 3′-C**Ds**C-5′, 3′-C**Ds**T-5′, 3′-T**Ds**C-5′ or 3′-G**Ds**G-5′ sequences (Figure 8). The yields for the pyrimidine-**Ds**-pyrimidine contexts of 3′-C**Ds**C-5′ and 3′-C**Ds**T-5′ were much higher than those for the purine-**Ds**-purine context of 3′-G**Ds**G-5′ in all substrates. We previously reported that the incorporation selectivity was also high (>95%) for the **Pa**TP and **Pa′**TP incorporations, using short DNA templates containing a 3′-C**Ds**T-5′ sequence [6]. However, **Pa**TPs modified with large molecules exhibited very low incorporation efficiencies even with 3′-T**Ds**C-5′, besides 3′-G**Ds**G-5′.

Taken together, the incorporations of **Pa** and modified-**Pa**, as well as those of **Pn**, close to the 3′-terminus of the transcript exhibited lower efficiencies, relative to those of the internal incorporations, as shown in Figure 3.

### 2.5. DNA Template Preparation for the **Ds**–**Pa** Transcription System

**Figure 9:**
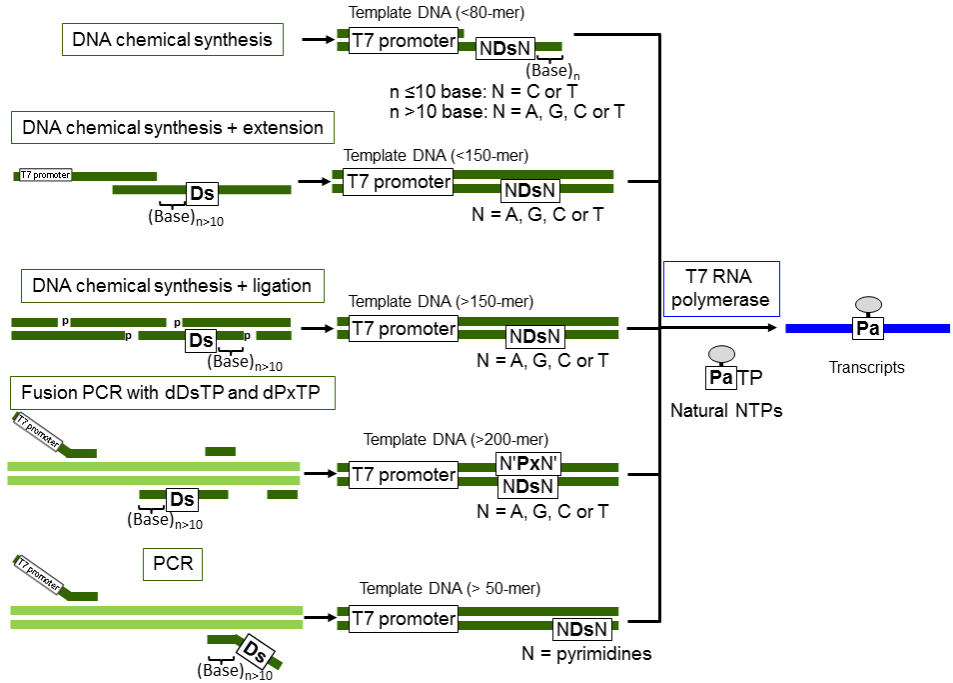
Schemes for the preparation of **Ds**-containing templates for T7 transcription, using **Ds**-containing DNA fragments.

For the practical uses of the **Ds**–**Pa** transcription system demonstrated here, the preparation methods for long **Ds**-containing templates are an important consideration. The general schemes for **Ds**-containing template preparation and the specific transcription of modified-**Pa**TPs are summarized in Figure 9. For the modified-**Pa**TP incorporation, **Ds**-containing DNA templates can be prepared by several methods. Templates (less than 80-mer including the T7 promoter) are prepared by a direct chemical method using a DNA synthesizer, as shown here. In this case, partially double stranded DNA templates for the promoter region can be used. Templates (more than 100-mer) can be prepared by several methods, as follows: (1) Chemical synthesis + enzymatic ligation: Chemically synthesized 5′-phosphorylated DNA fragments are annealed as a double stranded form and ligated by T4 DNA ligase [6,15]. As the complementary position opposite **Ds**, T can be used instead of the complementary unnatural base, **Px**. Phosphorylation of DNA fragments is performed during DNA chemical synthesis by using commercially available phosphorylation amidite reagents or T4 polynucleotide kinase. (2) Fusion PCR: For the internal introduction of **Ds** in long templates (more than 200-mer), fusion PCR methods involving the **Ds**–**Px** pair can be used with d**Ds**TP and d**Px**TP (unpublished data, Kimoto and Hirao). (3) PCR: For the terminal incorporation of **Ds** close to the 3′-terminus for transcripts, PCR amplification with primers containing **Ds** is useful [15,25]. In these three methods, the position of **Ds** introduction into each DNA fragment should be more than 10 bases away from the terminus.

## 3. Experimental 

### 3.1. General

Reagents and solvents were purchased from standard suppliers without further purification. Reactions were monitored by thin-layer chromatography (TLC), using 0.25 mm silica gel 60 plates impregnated with 254 nm fluorescent indicator (Merck). ^1^H-NMR, ^13^C-NMR, and ^31^P-NMR spectra were recorded on a Bruker (300-AVM) magnetic resonance spectrometer. Nucleoside purification was performed on a Gilson HPLC system with a preparative C18 column (Waters μ-BONDASPHERE, 150 × 19 mm). The triphosphate derivatives were purified by DEAE-Sephadex A-25 column chromatography (300 × 15 mm; eluted by a linear gradient of 50 mM to 1 M triethylammonium bicarbonate) and by HPLC with using a C18 column (CAPCELL PAK, 250 × 4.6 mm, SHISEIDO), eluted with a linear gradient of CH_3_CN in 100 mM triethylammonium acetate, pH 7.0. High resolution mass spectra (HRMS) and electrospray ionization mass spectra (ESI-MS) were recorded on a JEOL JM 700 or GC mate mass spectrometer and a Waters micromass ZMD 4000 equipped with a Waters 2690 LC system or a Waters UPLC-MS (H class) system, respectively. DNA templates were synthesized with an Applied Biosystems 392 DNA synthesizer, using CE phosphoramidite reagents for the natural and **Ds** bases (Glen Research), and were purified by gel electrophoresis. The syntheses of **Pa**TP, **Pa**′TP, NH_2_-**Pa**TP, and Biotin-**Pa**TP were described previously [6]. Biotin-**Pa**TP is commercially available from Glen Research, and the other modified-**Pa**TPs are available from TagCyx Biotechnologies. T7 RNA polymerase and T4 polynucleotide kinase were purchased from Takara. Antarctic phosphatase and streptavidin magnetic beads were purchased from New England Biolabs. The natural NTP Set (100 mM solutions: ATP, CTP, GTP, and UTP) and RNase T1 were purchased from GE Healthcare. The [γ-^32^P]GTP and [γ-^32^P]ATP were purchased from PerkinElmer. The *Escherichia coli* tRNA mixture was purchased from Sigma. Gel images were analyzed with a bio-imaging analyzer, FLA7000 (Fuji Film).

### 3.2. Synthesis of NH_2_-C3-**Pa**TP *(**4**)*

*1-(2,3-(Di-O-acetyl)-5-O-dimethoxytrityl-β-d-ribofuranosyl)-4-iodopyrrole-2-carbaldehyde* (**2**). 1-(β-d-Ribofuranosyl)-4-iodopyrrole-2-carbaldehyde (**1**) [6] (945 mg, 2.7 mmol) was dissolved in pyridine and evaporated to dryness *in vacuo*. The residue was dissolved in pyridine (27 mL), and 4,4′-dimethoxytrityl chloride (998 mg, 2.9 mmol) was added to the solution. The reaction mixture was stirred at room temperature for 1 h. After the reaction, the mixture was diluted with ethyl acetate (EtOAc) and washed with 5% NaHCO_3_ and brine. The organic phase was dried over Na_2_SO_4_, and evaporated *in vacuo*.

1-(5-*O*-Dimethoxytrityl-β-d-ribofuranosyl)-4-iodopyrrole-2-carbaldehyde (1.7 g) was purified by silica gel column chromatography (1% MeOH in CH_2_Cl_2_), and 2.6 mmol (1.7 g) of the purified product was co-evaporated with dry pyridine three times. The residue was dissolved in pyridine (26 mL), and acetic anhydride (979 μL, 10.4 mmol) was added to the solution. The mixture was stirred at room temperature for 12 h. After the reaction, the solution was diluted with EtOAc and washed with a saturated NaHCO_3_ solution. The organic layer was washed with brine, dried over MgSO_4_, and evaporated *in vacuo*. The product was purified by silica gel column chromatography in CH_2_Cl_2_, to give 1.77 g of **2** (89% yield from **1**). ^1^H-NMR (300 MHz, DMSO-*d*_6_) δ 9.49 (s, 1H), 7.69 (s, 1H), 7.39–7.24 (m, 10H), 6.89 (d, 4H, *J* = 7.8 Hz), 6.63 (d, 1H, *J* = 4.9 Hz), 5.52–5.42 (m, 2H), 4.25 (m, 1H), 3.74 (s, 6H), 3.39–3.25 (m, 2H), 2.05 (s, 3H), 2.04 (s, 3H).

*1-(2,3-(Di-O-acetyl)-β-d-ribofuranosyl)-4-(5-trifluoroacetamide-1-pentynyl)pyrrole-2-carbaldehyde* (**3**). The protected nucleoside **2** (148 mg, 0.2 mmol) was dissolved in DMF (1 mL) with CuI (6.1 mg, 0.032 mmol), Pd(PPh_3_)_4_ (11.6 mg, 0.01 mmol), and triethylamine (42 μL). To the solution, 5-trifluoroacetamide-1-pentyn (53.7 mg, 0.3 mmol) in DMF (0.6 mL) was added, and the solution was stirred at room temperature for 12 h. After the reaction, the mixture was diluted with EtOAc and washed with 5% NaHCO_3_ and brine. The organic phase was dried over Na_2_SO_4_ and evaporated *in vacuo*. The product was purified by silica gel column chromatography to give 1-(2,3-(di-*O*-acetyl)-5-*O*-dimethoxytrityl-β-d-ribofuranosyl)-4-(5-trifluoroacetamide-1-pentynyl)pyrrole-2-carbaldehyde. To the residue in CH_2_Cl_2_ (20 mL), 200 μL of dichloroacetic acid was added, and the solution was stirred at 0 °C for 15 min. The reaction mixture was poured into a saturated NaHCO_3_ solution, and the product was extracted with CH_2_Cl_2_. The combined organic layer was dried over MgSO_4_, evaporated *in vacuo*, and purified by silica gel column chromatography (1% MeOH in CH_2_Cl_2_), to give 83 mg of **3** (85% yield from **2**). ^1^H-NMR (300 MHz, DMSO-*d*_6_) δ 9.50 (s, 1H), 9.46 (bs, 1H), 7.98 (s, 1H), 7.18 (d, 1H, *J* = 1.7 Hz), 7.66 (d, 1H, *J* = 4.8 Hz), 5.41 (t, 1H, *J* = 5.1 Hz), 5.38–5.31 (m, 2H), 4.18 (m, 1H), 3.77–3.60 (m, 2H), 3.33–3.27 (m, 2H), 2.42 (t, 1H, *J* = 7.1 Hz), 2.08 (s, 3H), 2.02 (s, 3H), 1.76 (m, 2H).

*1-(β-d-Ribofuranosyl)-4-(5-amino-1-pentynyl)pyrrole-2-carbaldehyde 5′-triphosphate* (**4**). The protected nucleoside **3** (49 mg, 0.1 mmol) was dissolved in pyridine and evaporated to dryness *in vacuo*. The residue was dissolved in pyridine (100 μL) and dioxane (300 μL). A 1 M solution of 2-chloro-4*H*-1,3,2-benzodioxaphosphorin-4-one in dioxane (110 μL, 0.11 mmol) was added. After 10 min, tri-*n*-butylamine (100 μL) and 0.5 M bis(tributylammonium)pyrophosphate in DMF (300 μL, 0.15 mmol) were added to the reaction mixture. The mixture was stirred at room temperature for 10 min. A solution of 1% iodine in pyridine/water (98:2, v/v) (2.0 mL) was then added. After 15 min, a 5% aqueous solution (150 μL) of NaHSO_3_ was added. The volatile components were removed by evaporation, and then water (5 mL) was added to the residue. The solution was stirred at room temperature for 30 min, and then was treated with concentrated NH_4_OH (20 mL) at room temperature for 6 h. The solution was evaporated *in vacuo*, and the product was purified by DEAE-Sephadex A-25 column chromatography and C18 HPLC to give the nucleoside 5′-triphosphate **4** (NH_2_-C3-**Pa**TP), in a 31% yield from **3**. ^1^H-NMR (300 MHz, D_2_O) δ 9.36 (s, 1H), 8.04 (s, 1H), 7.16 (s, 1H), 6.48 (d, 1H, *J* = 2.9 Hz), 4.44 (m, 1H), 4.32–4.24 (m, 4H), 3.16 (m 2H), 3.15 (q, 22H, *J* = 7.3 Hz, TEA), 2.50 (t, 2H, *J* = 6.4 Hz), 1.89 (m, 2H), 1.23 (t, 32H, *J* = 7.3 Hz, TEA). ^31^P-NMR (121 MHz, D_2_O) δ −7.42 (bs, 1P), −10.39 (d, 1P, *J* = 17.2 Hz), −21.73 (t, 1P, *J* = 18.8 and 18.9 Hz). ESI-MS for C_15_H_23_O_14_N_2_P_3_ [M−H]^−^: calcd., 547.03; found, 546.82. UV-vis spectrum (in 10 mM sodium phosphate buffer, pH 7.0): λ_max_ 258 nm (ε = 12,200), 311 nm (ε = 10,100).

### 3.3. Synthesis of NH_2_-hx-**Pa**TP (**7**), FAM-hx-**Pa**TP (**8a**), and TAMRA-hx-**Pa**TP (**8b**)

*1-(β-d-Ribofuranosyl)-4-[3-(6-trifluoroacetamidohexanamido)-1-propynyl]pyrrole-2-carbaldehyde* (**5**). The nucleoside **1** (177 mg, 500 μmol, containing an α anomer) was co-evaporated with dry CH_3_CN twice. The residue was dissolved in DMF (2.5 mL) with CuI (15 mg, 80 μmol), Pd(PPh_3_)_4_ (29 mg, 25 μmol), and triethylamine (105 μL, 750 μmol), and the mixture was stirred in the dark at room temperature. To the solution, *N*-(2-propynyl)-6-trifluoroacetamidohexanamide (198 mg, 750 μmol) in DMF (2 mL) was added, and the solution was stirred at room temperature overnight. The reaction mixture was concentrated *in vacuo*. The product was purified from the residue by silica gel column chromatography (10% CH_3_OH in CH_2_Cl_2_) and C18 HPLC, to give 126 mg of **5** (51% yield). ^1^H-NMR (270 MHz, DMSO-d_6_) δ 9.52 (d, 1H, *J* = 0.66 Hz), 9.38 (brs, 1H), 8.27 (t, 1H, *J* = 5.3 Hz), 7.96 (s, 1H), 7.13 (d, 1H, *J* = 1.6 Hz), 6.32 (d, 1H, *J* = 3.6 Hz), 5.42–5.32 (brs, 1H), 5.18–5.05 (m, 2H), 4.08–3.95 (m, 4H), 3.90–3.84 (m, 1H), 3.70–3.50 (m, 2H), 3.20–3.10 (m, 2H), 2.08 (t, 2H, *J* = 7.3 Hz), 1.55–1.40 (m, 4H), 1.30–1.15 (m, 2H). HRMS (FAB, 3-NBA matrix) for C_21_H_27_F_3_N_3_O_7_ [M+H]^+^: calcd, 490.1801; found, 490.1815.

*1-(2,3-Di-O-acetyl-β-d-ribofuranosyl)-4-[(3-(6-trifluoroacetamidohexanamido)-1-propynyl]pyrrole-2-carbaldehyde* (**6**). The nucleoside **5** (122 mg, 250 μmol) was co-evaporated with dry pyridine three times. The residue was dissolved in pyridine (4 mL) with 4,4′-dimethoxytrityl chloride (89 mg, 263 μmol), and the solution was stirred at room temperature for 2.5 h. The solution was separated by EtOAc and water. The organic layer was washed with a saturated NaHCO_3_ solution, dried with MgSO_4_, and evaporated *in vacuo*. The residue was purified by silica gel column chromatography (0–0.5% CH_3_OH in CH_2_Cl_2_), to give 1-(5-*O*-dimethoxytrityl-β-d-ribofuranosyl)-4-[(3-(6-trifluoro- acetamidohexanamido)-1-propynyl]pyrrole-2-carbaldehyde (150 mg). The dimethoxytrityl derivative (149 mg, 188 μmol) was co-evaporated with dry pyridine three times. To the residue in pyridine (2 mL), acetic anhydride (53 μL, 565 μmol) was added. The mixture was stirred at room temperature overnight. The solution was separated by EtOAc and a saturated NaHCO_3_ solution. The organic layer was washed with brine, dried with MgSO_4_, and evaporated *in vacuo*. To the residue in dichloromethane (19 mL), dichloroacetic acid (280 μL) was added, and the solution was stirred at 0 °C for 15 min. The reaction mixture was poured into a saturated NaHCO_3_ solution and extracted with CH_2_Cl_2_. The combined organic layer was dried with MgSO_4_ and evaporated *in vacuo*. The product was purified by silica gel column chromatography (1–2.5% CH_3_OH in CH_2_Cl_2_), to give 90 mg of **6** (63% yield from **5**). ^1^H-NMR (270 MHz, DMSO-*d*_6_) δ 9.49 (s, 1H), 9.39 (brs, 1H), 8.28 (t, 1H, *J* = 5.3 Hz), 8.03 (s, 1H), 7.21 (d, 1H, *J* = 1.6 Hz), 6.63 (d, 1H, *J* = 5.3 Hz), 5.44 (brs, 1H), 5.40–5.28 (m, 3H), 4.20–4.15 (m, 1H), 4.05 (d, 2H, *J* = 5.3 Hz), 3.80–3.56 (m, 2H), 3.20–3.10 (m, 2H), 2.14–1.98 (m, 8H), 1.55–1.40 (m, 4H), 1.30–1.15 (m, 2H). HRMS (FAB, 3-NBA matrix) for C_25_H_31_F_3_N_3_O_9_ [M+H]^+^: calcd., 574.2012; found, 574.2061.

*1-(β-d-Ribofuranosyl)-4-[3-(6-aminohexanamido)-1-propynyl]pyrrole-2-carbaldehyde 5′-triphosphate* (**7**). The protected nucleoside **6** (57 mg, 100 μmol) was co-evaporated with dry pyridine three times. The residue was dissolved in pyridine (100 μL) and dioxane (300 μL). A 1 M solution of 2-chloro-4*H*-1,3,2-benzodioxaphosphorin-4-one in dioxane (110 μL, 110 μmol) was added to the solution, and then the reaction mixture was stirred at room temperature for 10 min. Aliquots of tri-*n*-butylamine (100 μL) and 0.5 M bis(tri-*n*-butylammonium)pyrophosphate in DMF (300 μL) were then added to the reaction mixture. After 10 min, a solution of 1% iodine in pyridine/water (98/2, v/v) (2.0 mL) was added and stirred. After 15 min, a 5% aqueous solution (150 μL) of NaHSO_3_ was added. The volatile components were removed by evaporation *in vacuo*, and the residue was dissolved in water (5 mL). The solution was stirred at room temperature for 30 min, and then was treated with concentrated ammonia (20 mL) at room temperature for 8 h. The solution was lyophilized, and the product was purified by DEAE-Sephadex A-25 column chromatography to give the nucleoside 5′-triphosphate **7** (NH_2_-hx-**Pa**TP), used for modification with a fluorophore, as described below. For further use as a transcription substrate, the triphosphate was purified by C18 HPLC. ^1^H-NMR (300 MHz, D_2_O) δ 9.39 (s, 1H), 7.93 (s, 1H), 7.21 (d, 1H, *J* = 1.1 Hz), 6.49 (d, 1H, *J* = 4.0 Hz), 4.38 (m, 2H), 4.23 (m, 3H), 4.10 (s, 2H), 3.15 (q, 14H, *J* = 7.3 Hz, TEA), 2.91 (t, 2H, *J* = 7.5 Hz), 2.26 (t, 2H, *J* = 7.1 Hz), 1.63 (m, 4H), 1.35 (m, 2H), 1.23 (t, 21H, *J* = 7.3 Hz, TEA). ^31^P-NMR (121 MHz, D_2_O) δ −9.04 (1P), −11.13 (d, 1P, *J* = 19.0 Hz), −22.66 (t, 1P, *J* = 19.8 Hz). ESI-MS for C_19_H_29_N_3_O_15_P_3_ [M−H]^−^: calcd., 632.08; found, 631.72. UV-vis spectrum (in 10 mM sodium phosphate buffer, pH 7.0): *λ*_max_ 308 nm (*ε* = 9,200).

*1-(β-d-Ribofuranosyl)-4-[3-[6-(fluorescein-5-carboxamido)hexanamido]-1-propynyl]pyrrole-2-carbaldehyde 5′- triphosphate* (**8a**) and 1*-(β-d-Ribofuranosyl)-4-[3-[6-(tetramethylrhodamine-5-carboxamido)hexanamido]-1-propynyl]pyrrole-2-carbaldehyde 5′-triphosphate* (**8b**).

(1) FAM-hx-**Pa**TP (**8a**): A 0.1 M NaHCO_3_-Na_2_CO_3_ buffer solution (pH 8.5, 2.5 mL) of **7** (one-third of the crude product) was reacted with 5-carboxyfluorescein *N*-hydroxysuccinimidyl ester (FAM-SE, Invitrogen) (9 mg, 19 μmol) in DMF (200 μL), in the dark at room temperature. After 9 h, the reaction was quenched with concentrated ammonia (1 mL). The product was purified by DEAE-Sephadex A-25 column chromatography and C18 HPLC to give **8a** (FAM-hx-**Pa**TP). ^1^H-NMR (300 MHz, D_2_O): δ 9.07 (s, 1H), 8.20 (s, 1H), 7.93 (d, 1H, *J* = 8.0 Hz), 7.62 (s, 1H), 7.13 (d, 1H, *J* = 7.9 Hz), 6.92 (d, 1H, *J* = 8.9 Hz), 6.89 (d, 1H, *J* = 9.0 Hz), 6.77 (m, 3H), 6.67 (d, 1H, *J* = 9.0 Hz), 6.64 (d, 1H, *J* = 9.2 Hz), 6.10 (d, 1H, *J* = 3.2 Hz), 4.14–3.93 (m, 5H), 3.87 (s, 2H), 3.32 (m, 2H), 3.04 (q, 21H, *J* = 7.3 Hz, TEA), 2.16 (t, 2H, *J* = 6.5 Hz), 1.54 (m, 4H), 1.35–1.09 (m, 35H, TEA). ^31^P-NMR (121 MHz, D_2_O) δ −10.90 (1P), −11.38 (1P), −23.25 (1P). ESI-MS for C_40_H_39_N_3_O_21_P_3_ [M−H]^−^: calcd., 990.13; found, 989.78. UV-vis spectrum (in 10 mM sodium phosphate buffer, pH 7.0): *λ*_max_ 493 nm (*ε* = 64,500).

(2) TAMRA-hx-**Pa**TP (**8b**): A 0.1 M NaHCO_3_-Na_2_CO_3_ buffer solution (pH 8.5, 2.5 mL) of **7** (one-third of the crude product) was reacted with 5-carboxytetramethylrhodamine *N*-hydroxysuccinimidyl ester (TAMRA-SE, Invitrogen) (10 mg, 19 μmol) in DMF (1 mL), in the dark at room temperature. After 8 h, the reaction was quenched with concentrated ammonia (1 mL) for 1 h. The product was purified by DEAE-Sephadex A-25 column chromatography and C18 HPLC to give **8b** (TAMRA-hx-**Pa**TP). ^1^H-NMR (300 MHz, D_2_O) δ 9.07 (s, 1H), 8.26 (s, 1H), 8.02 (d, 1H, *J* = 8.0 Hz), 7.69 (s, 1H), 7.35 (d, 1H, *J* = 7.9 Hz), 7.10 (d, 1H, *J* = 9.4 Hz), 7.01 (d, 1H, *J* = 9.4 Hz), 6.83–6.72 (m, 3H), 6.39 (d, 2H, *J* = 9.2 Hz), 6.06 (d, 1H, *J* = 3.5 Hz), 4.20 (t, 1H, *J* = 4.9 Hz), 4.05–3.95 (m, 6H), 3.42 (m, 2H), 3.16–3.07 (m, 33H, TEA), 2.25 (m, 2H), 1.63 (m, 4H), 1.40 (m, 2H), 1.20 (t, 28H, *J* = 7.3 Hz, TEA). ^31^P-NMR (121 MHz, D_2_O) δ −10.84 (d, 1P, *J* = 18.8 Hz), −11.56 (d, 1P, *J* = 17.5 Hz), −23.22 (t, 1P, *J* = 19.0 Hz). ESI-MS for C_44_H_49_N_5_O_19_P_3_ [M−H]^−^: calcd., 1044.22; found, 1044.09. UV-vis spectrum (in 10 mM sodium phosphate buffer, pH 7.0): *λ*_max_ 553 nm (*ε* = 107,000).

### 3.4. Synthesis of Dig-hx-**Pa**TP *(**10**)*

*1-(β-d-Ribofuranosyl)-4-[3-[6-(digoxigenin-3-O-methylcarbonyl)-aminohexananoyl]-amino-1-propynyl]pyrrole-2-carbaldehyde 5′-triphosphate* (**10**). A 0.1 M NaHCO_3_-Na_2_CO_3_ buffer solution (pH 8.5, 1 mL) of **9** (NH_2_-**Pa**TP, 8 μmol) was reacted with digoxigenin-3-*O*-methylcarbonyl-ε-aminocaproic acid-*N*-hydroxysuccinimide ester (Dig-hx-SE, Fluka) (5 mg, 7.6 μmol) in DMF (500 μL), at room temperature for 20 h. The product was purified by DEAE-Sephadex A-25 column chromatography and C18 HPLC, to give 5.3 μmol of **10** (Dig-hx-**Pa**TP) in a 70% yield. ^1^H-NMR (300 MHz, D_2_O) δ 9.39 (s, 1H), 7.88 (s, 1H), 7.21 (s, 1H), 6.47 (d, 1H, *J* = 4.0 Hz), 5.94 (s, 1H), 4.97 (s, 2H), 4.36 (m, 2H), 4.20 (m, 3H), 4.10 (s, 2H), 3.90 (s, 2H), 3.65 (s, 1H), 3.44 (m, 1H), 3.26–3.11 (m, 23H, TEA), 2.24 (t, 2H, *J* = 7.0 Hz), 2.19–1.90 (m, 2H), 1.85–1.38 (m, 19H), 1.23 (m, 36H, TEA), 0.89 (s, 3H), 0.71 (s, 3H). ^31^P-NMR (121 MHz, D_2_O) δ −10.69 (d, 1P, *J* = 18.8 Hz), −11.33 (d, 1P, *J* = 18.5 Hz), −23.13 (t, 1P, *J* = 19.7 Hz). ESI-MS for C_44_H_63_N_3_O_21_P_3_ [M−H]^−^: calcd., 1062.32; found, 1061.99. UV-vis spectrum (in 10 mM sodium phosphate buffer, pH 7.0): λ_max_ 308 nm (ε = 8,600).

### 3.5. DNA Templates for T7 Transcription

Each template DNA strand (10 μM; 65-mer, 60-mer, 55-mer, 50-mer, 45-mer, 42-mer, or 35-mer) was annealed with a 21-mer non-template strand (10 μM), in a buffer containing 10 mM Tris-HCl (pH 7.6) and 10 mM NaCl, by heating at 95 °C and slow cooling to 4 °C.

### 3.6. Transcription

Throughout the experiments, transcription (20 μL) was performed in a buffer containing 40 mM Tris–HCl (pH 8.0), 24 mM MgCl_2_, 5 mM DTT, 2 mM spermidine, and 0.01% Triton X-100, in the presence of 1 mM each NTP, 0 or 1 mM unnatural NTP, 0.05 μCi/μL [γ-^32^P]GTP, 2 μM DNA template, and 2.5 U/μL T7 RNA polymerase. After an incubation at 37 °C for 3 h, the reaction was quenched by adding an equivalent volume of a dye solution, containing 10 M urea and 0.05% BPB in 1× TBE. The reaction mixtures were heated at 75 °C for 3 min, and the products were analyzed on a 20% denaturing polyacrylamide gel containing 7 M urea, for the 17-mer transcripts, or a 10% denaturing polyacrylamide gel containing 7 M urea, for the other transcripts. 

### 3.7. Analysis of the Insertion Position of Biotin-**Pa** in the 48-Mer Transcripts

RNA transcripts (48-mer) with or without Biotin-**Pa** at position 21 were prepared by T7 transcription using a 65-mer DNA fragment as the template, and were purified by gel electrophoresis. After dephosphorylation by Antarctic phosphatase, each RNA (10 pmol) was labeled with ^32^P at the 5′-end by [γ-^32^P]ATP and T4 polynucleotide kinase, and was purified by gel electrophoresis. Each labeled RNA transcript (40,000 to 70,000 cpm) was dissolved in 20 μL of water. The labeled RNA was partially digested under alkaline conditions, by incubating the mixtures of the RNA solution (2 μL) and the H solution (5 μL, 50 mM sodium carbonate (pH 9.1) and 1 mM EDTA) for 10 min and 20 min at 90 °C respectively. The two digested samples (14 μL in total) and 6 μL of 1× PBS were combined and used for further treatment with streptavidin magnetic beads to capture the RNA fragments containing Biotin-**Pa**. As a sequence marker, we partially digested the labeled RNAs (1 μL) by mixing them with RNase T1 (1 μL, 1 U/μL) and the A solution (9 μL; 20 mM sodium citrate pH 5.0, 7 M urea, 1 mM EDTA, 0.025% BPB, and 0.25 mg/mL tRNA mixture), at 55 °C for 12 min. The partially-digested RNA solution in alkali (10 μL) was incubated with 20 μL of streptavidin magnetic beads (4 mg/mL) for 5 min at room temperature. We analyzed 5 μL aliquots of the supernatants and the other digested samples on a 10% denaturing polyacrylamide gel containing 7 M urea.

## 4. Conclusions

We have examined the site-specific incorporation of functional components into RNA by an unnatural base pair transcription system, using the **Ds**–**Pa** pairing. The **Pa** substrate can easily be chemically modified with a wide range of functional groups, via the propyne linker. These modified-**Pa**TPs can be site-specifically incorporated into RNA transcripts by conventional T7 transcription, by considering the characteristics of the **Ds**–**Pa** pair in transcription.

The **Pa**TPs modified with small molecules (MW less than ~240, such as **Pa**TP, **Pa**′TP, NH_2_-**Pa**TP, and Biotin-**Pa**TP) are efficiently incorporated into a specific internal position of a transcript, except for a position less than 10 bases from the 3′-terminus, without any natural base sequence dependency. For incorporation into a position close to the 3′-terminus of a transcript, the pyrimidine-**Pa**-pyrimidine sequences should be avoided, because of low transcription efficiency. Our previous results revealed that the incorporation selectivity of **Pa**TP opposite **Ds** in templates is more than 90%, and the misincorporation rate of **Pa**TP opposite the natural bases in templates is around 0.2% per base [6]. The present experiments using Biotin-**Pa**TP also displayed high selectivity (>90%).

The **Pa**TPs modified with large molecules (MW larger than ~480, such as TAMRA-hx-**Pa**TP, FAM-hx-**Pa**TP, and Dig-hx-**Pa**TP) exhibited reduced incorporation efficiency and selectivity in T7 transcription, and their incorporation into a position less than 10 bases from the 3′-terminus of the transcripts should be avoided. However, for their internal incorporation into RNA (less than ~50-mer), the intended transcripts can be isolated by gel electrophoresis, because the mobility of the transcripts containing the modified-**Pa** bases is significantly shifted on a gel, relative to that of transcripts lacking the modified-**Pa** bases.

This specific transcription involving the **Ds**–**Pa** pair could provide a useful tool for the site-specific introduction of functional groups of interest into RNA molecules with long chains. The incorporation of **Pa**TPs modified with large molecules, such as fluorescent dyes, is unfavorable for specific transcription, and thus, as an alternative method, the efficient incorporation of the NH_2_-**Pa** bases can be used as a site for post-transcriptional modifications with large functional molecules. 

## Figures and Tables

*Reagents and abbreviations*: (a) (i) 4,4′-dimethoxytrityl chloride, pyridine (ii) acetic anhydride, pyridine; (b) (i) CuI, tetrakis(triphenylphosphine)palladium, triethylamine, trifluoroacetamide-1-pentyne, DMF, (ii) dichloroacetic acid, dichloromethane; (c) 2-chloro-4*H*-1,3,2-benzodioxaphosphorin-4-one/dioxane, pyridine, tri-*n*-butylamine, bis(tributylammonium)pyrophosphate, DMF, then I_2_/pyridine, water, NaHSO_3_, H_2_O, NH_4_OH. DMTr: 4,4′-dimethoxytrityl, Ac: acetyl.

*Reagents and abbreviations*: (a) (i) CuI, tetrakis(triphenylphosphine)palladium, DMF, triethylamine; (ii) *N*-(2-propynyl)-6-trifluoroacetamidohexanamide; (b) 4,4′-dimethoxytrityl chloride, pyridine; (c) (i) acetic anhydride, pyridine, (ii) dichloroacetic acid, dichloromethane; (d) 2-chloro-4*H*-1,3,2-benzodioxaphosphorin-4-one/dioxane, pyridine, tri-*n*-butylamine, bis(tri-*n*-butylammonium)pyrophosphate, DMF, then I_2_/pyridine, water, NaHSO_3_, NH_4_OH; (e) R-*N*-hydroxysuccinimidyl ester (R = 5-carboxyfluorescein or 5-carboxytetramethylrhodamine)/DMF, 0.1 M NaHCO_3_-Na_2_CO_3_ buffer (pH 8.5), then NH_4_OH. Ac: acetyl.

*Reagents*: Dig-*N*-hydroxysuccinimidyl ester / DMF, 0.1 M NaHCO_3_-Na_2_CO_3_ buffer (pH 8.5).
